# Role of Appetite Loss and Inflammation on Incident Disability Among Community-Dwelling Older Adults

**DOI:** 10.1093/geroni/igaf122.4004

**Published:** 2025-12-31

**Authors:** Germine Soliman, Anne Newman, Shanshan Yao, Xiaomin Lu, George Kuchel, Lisa Barry

**Affiliations:** University of Connecticut, Farmington, Connecticut, United States; University of Pittsburgh, Pittsburgh, Pennsylvania, United States; University of Michigan, Ann Arbor, Michigan, United States; University of Connecticut, Farmington, Connecticut, United States; UCONN HEALTH, Farmington, Connecticut, United States; University of Connecticut, Farmington, Connecticut, United States

## Abstract

Aging is associated with reduced appetite and increased pro-inflammatory status, both of which may contribute to disability. We evaluated whether appetite loss and inflammatory markers were associated with incident disability in community-dwelling older adults. Health, Aging, and Body Composition Study participants (n = 3,071) were asked about their appetite in the past month, with responses dichotomized as good versus impaired. Participants were followed up to 6 years to determine if they experienced incident Persistent Lower Extremity Limitation (PLL), defined as two consecutive self-reports of difficulty walking a quarter mile and/or climbing 10 steps without resting. Biomarkers (IL6, TNF-α, CRP) were assessed at baseline. 388 (12.6%) individuals reported poor appetite, mainly female (59%), Black (54%), with a mean age of 73.9 (±3) years. A total of 64% reporting poor appetite and 47% reporting good appetite developed PLL (p < 0.001). Levels of IL6, TNF-α, and CRP were higher among those with poor appetite (p < 0.001 for each comparison). A Fine-Gray proportional hazards model adjusted for demographics, chronic diseases, anxiety, depression, prescribed medications, and sensory factors, indicated that poor appetite (hazard ratio[HR]= 1.56; 95% CI 1.29, 1.87) and inflammatory markers ((HR IL6=1.05; 95% CI 1.02, 1.09); (HR TNF-α =1.05; 95% CI 1.02, 1.08); (HR CRP=1.02; 95% CI 1.00, 1.04)) were each associated with developing PLL. Our findings show that reducing appetite loss and inflammation may be promising targets for minimizing disability in older adults. Additional research will assess the potential mediating role of inflammation in the relationship between appetite loss and disability.

